# Age- and trait-dependent breeding responses to environmental variation in a short-lived songbird

**DOI:** 10.1038/s41598-023-42166-2

**Published:** 2023-09-11

**Authors:** Rafał Martyka, Aneta Arct, Dorota Kotowska, Lars Gustafsson

**Affiliations:** 1 Animal Ecology, Department of Ecology and Genetics, Evolutionary Biology Centre, Uppsala University, Norbyvägen 18D, 752 36 Uppsala, Sweden; 2 Institute of Nature Conservation, Polish Academy of Sciences, Mickiewicza 33, 31-120 Kraków, Poland; 3 Institute of Systematics and Evolution of Animals, Polish Academy of Sciences, Sławkowska 17, 31-016 Kraków, Poland

**Keywords:** Evolutionary ecology, Evolution, Zoology

## Abstract

Breeding responses of organisms to environmental changes may profoundly depend on an individual’s age, as an age-environment interaction may be expected to affect the expression of reproductive traits. However, little is known about how this interaction affects short-lived species that experience various environmental conditions in adulthood. Here, we used a 32-year dataset from the collared flycatcher, *Ficedula albicollis,* population to test whether and how the environment interacts with age to shape female age-specific reproduction. To characterise environmental variation, we applied the remotely sensed normalised difference vegetation index (NDVI), estimating vegetation productivity, and used it as a surrogate for habitat quality. Then, we analysed how within-individual age and NDVI determine patterns in laying date, clutch size, offspring production, and recruitment. We found that young and old females, but not middle-aged females, breeding under low NDVI started to lay eggs later and produced smaller clutches than females of the same age breeding under higher NDVI. No such effects were observed for offspring production or recruitment. Our study provides evidence that both an individual’s age and the environmental variation experienced during adulthood may be crucial for shaping reproductive patterns in short-lived avian species, as has been found in long-lived birds.

## Introduction

Age-specific reproductive performance is a phenomenon widely observed in natural populations of animals and remains a key issue in population ecology^1–3^. In general, a common pattern is observed: reproductive performance increases at a young age (so-called early-life improvement) to achieve a peak in middle age, and then decreases in older age (so-called late-life decline or senescence)^1,3,4^. At the level of individuals, the early-life improvement in reproductive performance is considered to be a result of the acquired breeding experience with age, and/or reduced investment in first breeding attempts^3,5^. In turn, the within-individual decline in reproductive performance in advanced age is commonly attributed to the senescence process, resulting from the irreversible accumulation of damage that consequently leads to the loss of the organism’s function^4,6^. Thus, both early-life improvement and late-life senescence may be considered the main intrinsic processes that determine the observed age-related patterns of reproductive performance in animal natural populations.

Despite the significant role that intrinsic factors play in shaping age-related reproductive patterns, extrinsic drivers are also indicated to be crucial for age-dependent breeding performance. In this context, environmental variation and its interaction with age seem to be of the greatest importance^7^. Environmental stress, such as periods of low food availability, high predation, or increased habitat disturbance, is a significant driver in reducing fitness, and individuals in different age classes may vary in their responses to environmental conditions. In particular, middle-aged individuals may possess the necessary experience and resources required to adequately cope with environmental stochasticity in comparison to younger and older ones^8–10^. So far, the effects of interaction between age and environmental conditions on patterns of reproductive performance have been examined by several studies, with different patterns observed^9–19^. Most of them indicate that the age-environment interaction has the greatest impact on individuals in early adulthood. In fact, in a resource-poor environment, younger females of Tengmalm’s owl, *Aegolius funereus*, laid smaller clutches^12^; the Nazca boobies, *Sula granti*, at early life had a lower probability of breeding, delayed egg-laying initiation, and smaller clutches^10^; and young western gulls, *Larus occidentalis*, showed a decline in offspring production^11^. However, resource-poor environmental conditions experienced during adult life are also expected to negatively affect senescent birds^8^. Tompkins and Anderson^10^ indeed found that old females breeding under the challenging environmental conditions experienced over their adult lives showed a delayed breeding date and lower fledgling success. In turn, Reed et al.^14^ showed that poor environmental conditions experienced at an early stage of adult life decreased breeding success in late life. However, other studies did not confirm the effect of environmental variation experienced during adulthood on reproductive performance at an advanced individual’s age^13,15^, or even showed opposite patterns^9,16^. In contrast, the poor-quality natal (developmental) environment experienced by the offspring is a well-known driver accelerating the rate of reproductive senescence in adult life^17–19^. Existing evidence indicates that an interaction of age and environment seems to be significant for shaping patterns of reproductive performance in birds. However, past studies have mostly concentrated on exploring the age-dependent effects of the environment that were experienced in adult life among long-lived bird species^9–16^. Therefore, to fully understand the patterns of age-specific reproductive performance over a lifetime in an environmental context, we need to examine these relationships for taxa with various life history traits. Taking this into account, the interactive effects of age and environmental variation experienced in adulthood are clearly understudied in short-lived passerine birds.

Our aim was to determine whether and how environmental variation experienced in adult life affects age-specific reproductive performance in females of a short-lived passerine species. For this purpose, we used a 32-year-long dataset from the Gotland population of the collared flycatcher, *Ficedula albicollis*, which makes an excellent study system for our research due to the availability of data on the accurate age and reproductive performance of individuals. We assessed the reproductive performance based on four different reproductive traits: laying date, clutch size, offspring production (i.e., the number of produced offspring that leave the nest), and recruitment (i.e., the number of recruits and the probability of recruitment at brood level). Specifically, we examined the effect of within-individual age on those reproductive traits and investigated how environmental conditions modify age-dependent changes in reproductive performance. To determine variation in environmental conditions (environmental quality), we used the satellite-derived normalised difference vegetation index (NDVI), which is a measure of vegetation greenness and biomass commonly used to assess patterns of vegetation productivity at spatial and temporal scales^20^. Importantly, plant productivity index is used as a surrogate for habitat quality in studies on habitat selection, resource quality and space use, ecosystem functioning, or trophic interactions^21–23^, mainly because it is correlated with climatic conditions^24,25^, the abundance of herbivorous insects and mammals^26–28^, and the breeding performance of birds and mammals^29–31^. Using NDVI as a proxy for environmental conditions, we assume that higher biomass vegetation reflects higher prey abundance (i.e., food availability).

In passerine bird species, including the collared flycatcher, reproductive performance changes with the age of individuals, with performance increasing early in life and decreasing late in life^32–34^. Thus, we expected that the female’s age at the individual level would influence the examined reproductive traits, and their changes should have a non-linear (quadratic) pattern, with young and old females exhibiting poor reproductive performance compared to middle-aged females. However, poor-performing females, that is, those in the first and last age classes, may be especially vulnerable to low-quality environmental conditions^8,10–12,14^. This is because when environmental resources are scarce (e.g., food quality or availability), the reproductive costs for inexperienced or senescent individuals should be higher^35^. Therefore, we predicted that a relatively resource-poor environment (reflected in our study by low NDVI values) would enhance the effect of age on reproductive traits compared to a relatively resource-rich environment (reflected by high NDVI values). Thus, young and old females are expected to show a larger decline in reproductive performance in poor environmental conditions than in an environment of higher quality, which should be statistically reflected by the interaction of female age and NDVI.

## Results

### Effects of female age and environmental conditions (NDVI) on laying date

**Figure 1:**
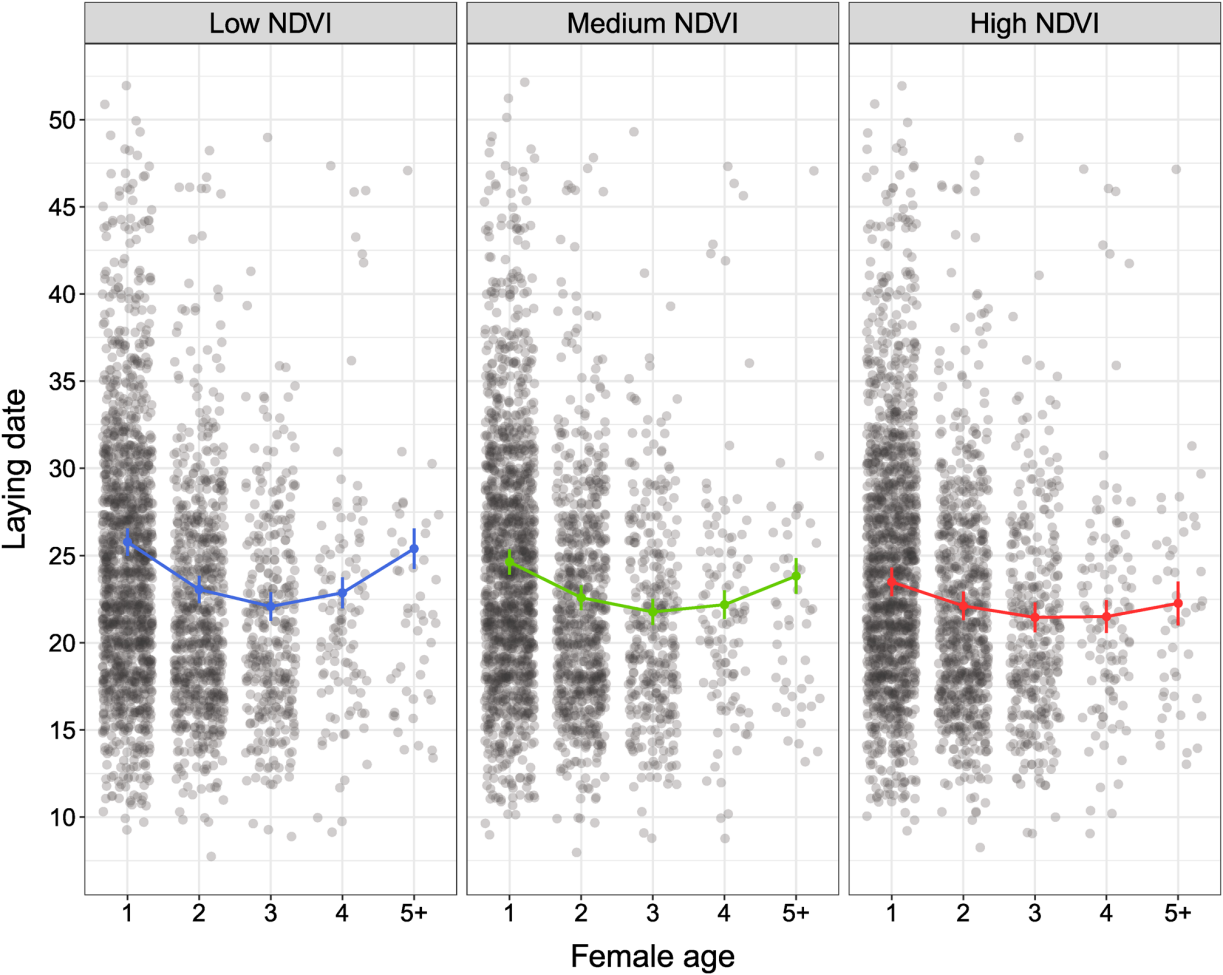
The quadratic effect of female age and environmental conditions (low, medium, and high NDVI) in relation to laying date. Predicted means with SEs (colours) obtained from the final model and raw data points (grey circles) are presented.

We found that variation in laying date was significantly explained by an interaction of the quadratic effect of female age and NDVI (Table 1). This interaction implies that females of young and old age breeding under low NDVI started to lay eggs later compared to females of the same age breeding under high NDVI (Table 1). Females in the same age classes but reproducing under middle NDVI showed an intermediate pattern (Fig. 1). In contrast to young and old females, middle-aged ones were not affected by distinct environmental conditions (Fig. 1). AFR and ALR had no effect on the initiation of breeding (Table 1), which suggests that the selective appearance and disappearance of individuals in the studied population do not influence variation in laying date.

**Table 1:** Results of final linear mixed models examining effects of age (as a linear and quadratic term), age at first reproduction (AFR), age at last reproduction (ALR), environmental conditions (NDVI), and an interaction of age (as a linear and quadratic term) and NDVI on laying date, clutch size, and the number of fledglings per female per year.

Source of variation	Response variables					
	Laying date (*N* = 2843 broods of 2058 females)		Clutch size (*N* = 2822 broods of 2044 females)		Fledgling number (*N* = 2422 broods of 1802 females)	
Intercept	**23.058**	(21.661, 24.456)***	**6.184**	(6.101, 6.266)***	**3.729**	(3.291, 4.066)***
Age	**− 1.687**	(− 2.090, − 1.283)***	**0.249**	(0.198, 0.301)***	**0.327**	(0.132, 0.522)**
Age^2^	**0.546**	(0.388, 0.704)***	**− 0.086**	(− 0.107, − 0.065)***	**− 0.157**	(− 0.234, − 0.080)***
AFR	− 0.130	(− 0.372, 0.113)	**− 0.038**	(− 0.072, − 0.003)*	0.001	(− 0.109, 0.111)
ALR	− 0.080	(− 0.395, 0.234)	0.001	(− 0.045, 0.043)	**0.203**	(0.059, 0.347)**
NDVI	− 0.613	(− 1.295, 0.069)**’**	0.055	(− 0.010, 0.121)	− 0.099	(− 0.344, 0.145)
Residuals_laying date model_	–	–	**− 0.294**	(− 0.320, − 0.268)***	–	–
Residuals_clutch size model_	–	–	–	–	**0.244**	(0.152, 0.335)***
Age × NDVI	**0.534**	(0.242, 0.826)***	**− 0.061**	(− 0.100, − 0.022)**	–	–
Age^2^ × NDVI	**− 0.231**	(− 0.380, − 0.081)**	**0.023**	(0.004, 0.043)*	–	–
Fixed terms_pooled_	2.616		0.198		0.197	
Female ID_random_	3.696		0.159		0.121	
Plot ID_random_	0.439		0.003		0.103	
Year_random_	14.165		0.037		0.561	
Residual	24.060		0.361		5.126	

In models analysing clutch size and fledgling number, residuals from laying date and clutch size models were entered as covariates to control for the timing of breeding and the number of produced eggs, respectively. To reduce models (if applicable), only non-significant interactions were removed if *P* > 0.10. For fixed terms, parameter estimates accompanied by 95% CIs are presented. Variance estimates for fixed terms (pooled), each random factor, and the residual component obtained from the final model are also shown. The significance level for fixed terms is coded as ***(*P* < 0.001), **(*P* < 0.01), *(*P* < 0.05), and ’(*P* < 0.10). Significant terms are in bold.

### Effects of female age and environmental conditions (NDVI) on clutch size

**Figure 2:**
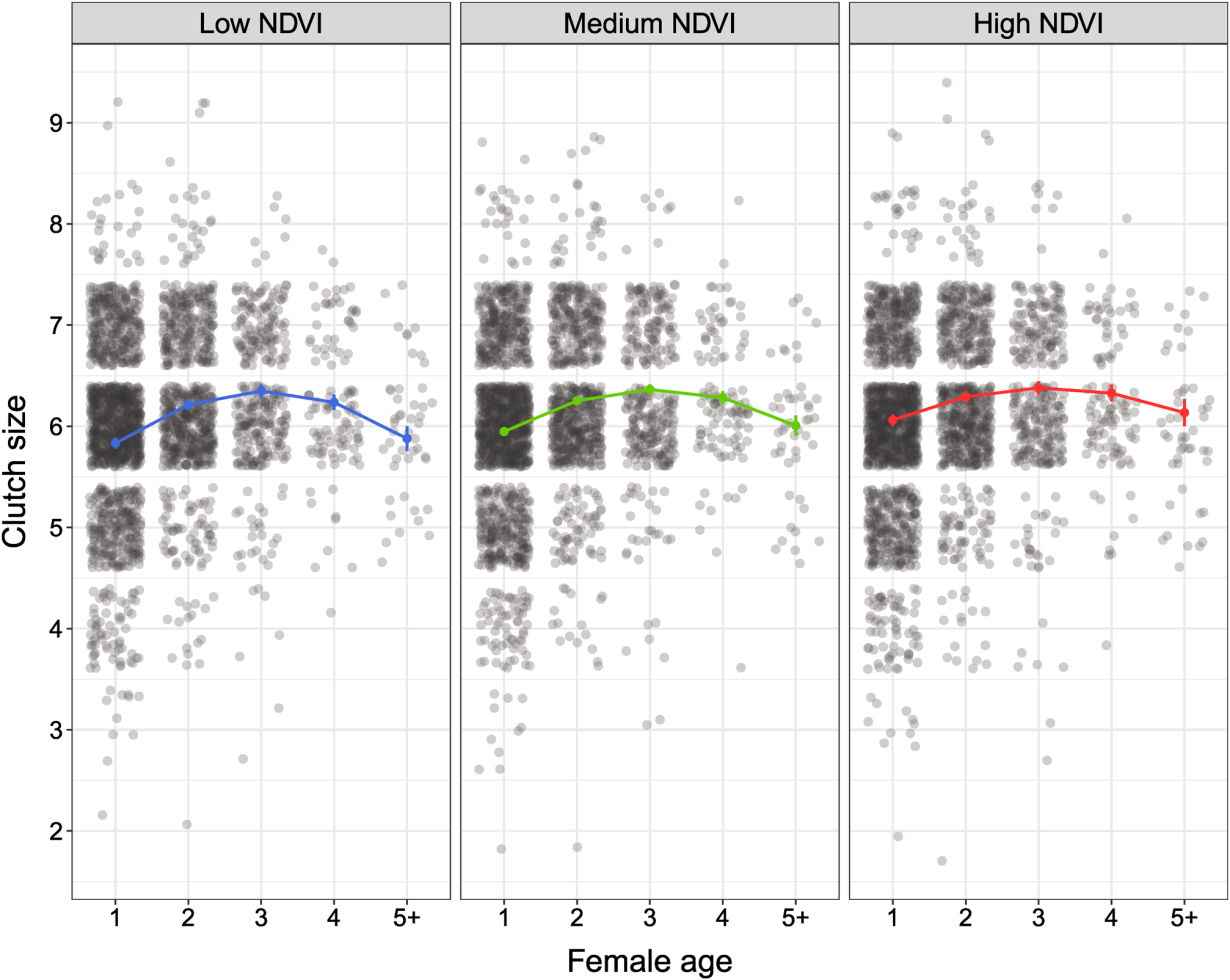
The quadratic effect of female age and environmental conditions (low, medium, and high NDVI) in relation to clutch size. Predicted means with SEs (colours) obtained from the final model and raw data points (grey circles) are presented.

Variation in clutch size was also significantly influenced by the interaction of the quadratic effect of female age and NDVI (Table 1). This interaction results from the fact that young and old females reproducing under low NDVI produced smaller clutches than the same females that experienced high NDVI (Fig. 2). In turn, females in the same age groups breeding under the middle NDVI showed an intermediate pattern (Table 2). Differences in environmental conditions did not influence middle-aged females (Fig. 2). We also found that AFR was negatively related to clutch size (Table 1), indicating that at the population level, females beginning to breed at a later age laid smaller clutches. Moreover, the number of laid eggs decreased with the advance of the breeding season, as revealed by the negative correlation between residuals from the laying date model and clutch size (Table 1). ALR did not affect this reproductive trait (Table 1).

**Table 2:** Results of final generalised linear mixed models, with Poisson error variance and log-link function and binomial error variance and logit-link function, examining effects of age (as a linear and quadratic term), age at first reproduction (AFR), age at last reproduction (ALR), environmental conditions (NDVI), and an interaction of age (as a linear and quadratic term) and NDVI on the number of recruits and recruitment probability per female per year.

Source of variation	Response variables			
	Recruit number (*N* = 2422 broods of 1802 females)		Recruitment probability (*N* = 2422 broods of 1802 females)	
Intercept	**− 1.366**	(− 1.602, − 1.129)***	**− 1.244**	(− 1.613, − 0.875)***
Age	0.112	(− 0.012, 0.236)’	0.187	(− 0.027, 0.400)’
Age^2^	**− 0.060**	(− 0.109, − 0.010)*	**− 0.095**	(− 0.181, − 0.009)*
AFR	− 0.034	(− 0.106, 0.038)	− 0.069	(− 0.193, 0.055)
ALR	**0.187**	(0.097, 0.278)***	**0.225**	(0.070, 0.381)**
NDVI	− 0.071	(− 0.223, 0.081)	− 0.117	(− 0.356, 0.122)
Residuals_fledgling number model_	**0.975**	(0.877, 1.074)***	**1.326**	(1.180, 1.471)***
Fixed terms_pooled_	1.001		1.844	
Female ID_random_	0.000		0.000	
Plot ID_random_	0.077		0.249	
Year_random_	0.192		0.422	
Residual	1.323		3.290	

Residuals from the fledgling number model were entered as a covariate to control for the number of produced offspring. To reduce models, only non-significant interactions were removed if *P* > 0.10. For fixed terms, parameter estimates accompanied by 95% CIs are presented. Variance estimates for fixed terms (pooled), each random factor, and the residual component obtained from the final model are also shown. The significance level for fixed terms is coded as ***(*P* < 0.001), **(*P* < 0.01), *(*P* < 0.05), and ’(*P* < 0.10). Significant terms are in bold.

### Effects of female age and environmental conditions (NDVI) on offspring production

The number of fledglings per nest was significantly affected by the quadratic effect of female age, ALR, and residuals from the clutch size model (Table 1). In turn, AFR and NDVI did not influence this reproductive trait (Table 1). The number of fledglings leaving the nest changed across female ages, with a pattern indicating an increase in offspring production in early life and a decrease in their production in late life, with middle-aged females showing a peak of performance (Figure S1). ALR positively influenced the number of fledglings (Table 1), which means that longer-lived females raised more offspring. There was a positive correlation between residuals from the clutch size model and offspring number, indicating that larger clutches produced more fledglings (Table 1).

### Effects of female age and environmental conditions (NDVI) on recruitment

We found that both the number of recruits and the probability of recruitment were significantly explained by the quadratic effect of female age, ALR, and residuals from the fledgling number model (Table 2). AFR and NDVI did not affect the number of recruited offspring or recruitment probability (Table 2). The recruitment pattern shows a slight increase at early female age, reaching its highest value in middle-aged females, and a slight decrease at old female age (Figure S2). ALR and residuals from the fledgling number model positively predicted offspring recruitment (Table 2), indicating that long-lived females and those with larger offspring production had more recruits and a higher recruitment probability.

## Discussion

Our study clearly shows that changes in reproductive traits at the level of individuals are dependent on age, and the observed age-specific effects on reproductive performance are represented by non-linear (quadratic) patterns. As we expected, this is determined by the fact that middle-aged females showed the highest reproductive performance across all examined reproductive traits compared to younger and older females, whose reproductive parameters were deteriorated (we interpret the main effect of age for models with age × NDVI interaction, such as in models without this interaction, due to the character of that interaction: the age effect is the same across various NDVI, and the only difference is the degree to which reproductive traits of young and old females change in response to experienced NDVI). Specifically, young and older females started laying eggs later, produced smaller clutches, and had fewer offspring and recruits. In contrast, females in the middle age classes laid eggs earlier, had larger clutches, and produced more offspring and recruits. Consequently, our findings indicate a process of early-life improvement and late-life decline in the examined reproductive traits. The early-life improvement in reproductive performance may result from the acquisition of experience during first breeding attempts^3^, as was confirmed in an experimental study on the collared flycatcher^36^, and/or an optimisation of reproductive effort^5,32^. In turn, reproductive senescence in older age classes is a common process observed in natural populations^37^. Due to the intrinsic deterioration in physiological processes and cellular functioning in old age, individuals may experience a reduction in survival or breeding performance^4^. The important factors that affect the rate of reproductive aging are early-life reproductive effort^14,32^ and environmental circumstances^4,6^. Both the increase in reproductive performance in early life and the reproductive senescence in late life have been previously documented in several bird species^10,33,34^, including the collared flycatcher^32^. We documented that birds reached their peak of performance in the examined reproductive traits at approximately the age of three years. This is in correspondence with earlier studies on the pied and collared flycatchers and also other short-lived passerine species, which, similarly to our research, showed differences in reproductive performance between breeders in the first and last age classes and those in intermediate age^32,34,38,39^.

In comparison to within-female effects represented by the squared individual's age, which in a similar manner explained variation in reproductive traits, between-female effects represented by AFR and ALR showed different patterns across the examined traits. ALR influenced only offspring production and recruitment, with no effects on laying date or clutch size. ALR positively predicted the number of fledglings and recruits, or recruitment probability. This means that at the population level, both reproductive parameters are determined by individuals’ lifespan, which implies that females who live longer produce more offspring and recruits. This suggests that offspring production and recruitment, but not laying date and clutch size, are probably driven by the selective disappearance of individuals with low phenotypic quality. Indeed, high-quality birds usually live longer than low-quality individuals, and thus they may achieve higher reproductive success. In turn, AFR significantly affected only clutch size, without any effects on remaining reproductive traits. A negative influence of AFR on the number of eggs laid in a clutch indicates that at the population level, females starting to breed for the first time at a later age produced smaller clutches. This means that only maternal investment in a clutch seems to be associated with the selective appearance of females in that population.

The crucial finding of our study is that reproductive performance is not only shaped by intrinsic factors related to age but may also be affected by extrinsic environmental conditions experienced by individuals during their adult lives. We showed that the age-specific patterns of two out of four examined reproductive traits, i.e., laying date and clutch size, were modified by variation in the NDVI. In the case of laying date, young and old females experiencing relatively poor environmental conditions (low NDVI) exhibited lower performance, i.e., delayed egg-laying initiation, than females of the same age breeding in relatively good environmental conditions (high NDVI). In turn, middle-aged females that started to lay eggs the earliest coped evenly well across different environmental conditions (from low to high NDVI). We observed a similar pattern in the case of clutch size: young and old females produced smaller clutches when experiencing low NDVI compared to females breeding under high NDVI. Again, middle-aged females that laid the largest clutches, performed similarly in different environmental conditions (from low to high NDVI). These results are in agreement with our predictions that young and old breeders may suffer from inexperience and aging, respectively, and therefore may be especially affected by poor environmental conditions. In fact, a resource-poor environment, such as that represented by a lower NDVI, may generate higher reproductive effort or increased maternal investment in young breeders than in more experienced birds. Thus, young and inexperienced birds may reduce reproductive performance under poor environmental conditions to decrease the costs of early-life reproductive effort. Such mechanism may allow them to increase investment in subsequent breeding attempts, e.g., when environmental conditions are better or when the individual is more experienced and thus is able to cope better with adverse conditions. In the case of old females, we observed that when they bred under poor environmental conditions, they experienced reproductive senescence at higher rate. This implies that old individuals, due to aging processes, may incur higher costs of self-maintenance in a poor-resource environment compared to a rich-resource one, which consequently results in declining investment in reproduction.

The fact that environmental conditions, especially those experienced during early life (including the developmental environment), impose great effects on the rate of reproductive aging, has already been recognised^6,19^. Indeed, previous research on adult long-lived birds has shown that individual’s age and environmental conditions may interact to affect reproductive traits such as breeding date, clutch size, or offspring production^10–12,14^. However, the majority of those studies documented that environmental conditions influence mostly young birds but not those in the middle or older age classes. This leads to the conclusion that environmental variation may play an especially important role in shaping the patterns of early-life improvement in breeding performance^10^. In fact, the results of our research fit into this picture. In contrast, to date, only a few studies have documented that environmental variation experienced during an individual’s adult life influences the rate of reproductive senescence in old age^10,14^. Our study significantly contributes to existing knowledge by showing that variation in environmental conditions encountered across an adult’s lifespan may indeed be an important driver affecting reproductive senescence in late life. However, both the results of previous studies and our research indicate that the patterns of age-by-environment interactions are generally complex and may be non-uniform across reproductive traits, species characteristics, studied populations, or proxies used for the determination of environmental conditions.

To conclude, we demonstrated that the environment may interact with the age of individuals to affect their reproductive performance. Further, we provided evidence that environmental conditions experienced during adult life may also play a significant role in shaping age-specific reproductive patterns in short-lived avian species, as has previously been documented in long-lived birds. Specifically, we demonstrated that young and old females but not middle-aged ones breeding in poor-resource environments exhibit lower reproductive performance in terms of laying date and clutch size. Our study also highlighted that to effectively examine the causes and consequences of age-related variation in reproductive performance, one needs to account for the environment experienced by adult individuals.

## Methods

### Ethics statement

The study uses the data that has been previously collected during the long-term study in a wild population of collared flycatchers, but we confirm that all procedures and experiments conducted during the longitudinal research were performed in accordance with relevant guidelines and regulations (according to Swedish law).

### Study population and data collection

Our research spans the years 1986–2016 and is part of a long-term study conducted in a nest-box breeding population of the collared flycatcher, *Ficedula albicollis*, on the Swedish island of Gotland (57° 10′ N, 18° 20′ E). Collared flycatchers are small (ca. 13 g of weight), migratory, and hole-nesting passerine bird species inhabiting deciduous and mixed forests of eastern, central, and south Europe and south-western Asia, with single and isolated northern populations^40^. In the studied northern population, females commonly lay 4–8 eggs in one breeding event per year. Incubation lasts ca.14 days, and nestlings remain in the nest for 14–16 days, where they are fed by both parents^41^. Most males are socially monogamous, but alternative mating strategies are also quite frequent; socially polygynous males represent up to 9% of all males^42^, and the percentage of males involved in extra-pair copulations is even higher and constitutes up to 15.5% of all nestlings sired by extra-pair males^43,44^.

The Gotland population of the collared flycatcher is appropriate to study the age-specific pattern in reproductive traits because of specific features of this population such as preferring breeding in nest boxes over natural cavities (such birds are observed occasionally and definitely do not exceed 5% of all breeders in most plots; L. Gustafsson, personal observation), the high probability of between-year site fidelity (approximately 0.8 for females), and limited natal dispersal of individuals (a median for females is 840 m)^45–47^. Moreover, unringed birds that are caught, aged, and marked for the first time constitute, on average, 20% of breeders each year^47^. This allows to collect data on the exact age of birds and monitor their lives year by year. Breeders are caught at nest boxes, in the case of females mostly during incubation, and in the case of males during nestling provisioning. The age of previously unringed individuals is classified dichotomically as first-year or older based on the shape of primary coverts and the colour of the upper mandible (in females) or the colour of flight feathers (in males)^39^. All birds are individually marked using aluminium rings. Moreover, detailed information on their reproductive parameters is gathered, especially about laying date, clutch size, hatching date, brood size, number of fledglings (offspring that left the nest), and recruits (offspring that were recruited to the breeding population). Also, nestlings are ringed and measured on day 12 after hatching (for further details about the population and methods, see Gustafsson^41^).

Collared flycatchers breed in wooden nest boxes located in many distinct nest-box areas (study plots), which are distributed in the southern part of the island of Gotland over an area extending about 16 km (north–south) and 8 km (east–west). Study plots are located in deciduous forests (i.e., woods dominated by oaks, ashes, and poplars, typically with dense understory dominated by common hazels and oneseed hawthorns), forest-meadow habitats (i.e., bright, sparse oak woods with rich hay meadows), and pine-dominated forests with admixture of deciduous trees^48^. In the current study, we used data collected from 24 study plots, mostly existing over the whole study period. The plots differed in the total area, the number of available nest boxes, habitat structure, and NDVI values (for more details on study plots, see Table S1).

### NDVI estimation

Data on NDVI was collected for the breeding seasons of 1984–2016, with May and June being the most important periods during the breeding of our model population. To generate multi-year NDVI time series for the whole period, we used all the available Landsat 5, 7, and 8 data products of Level 1 (i.e., radiometrically, atmospherically, and geometrically corrected satellite images at 30 m spatial resolution) covering the study area (all examined plots). Only scenes with less than 80% cloud cover (acquired in May and June) were chosen for analysis. The selected raster dataset was masked to exclude pixels containing clouds, water-saturated values, haze, and aerosol interference based on quality assurance bands included in the images. For each pixel of each scene, the NDVI value was calculated and then calibrated to avoid cross-sensor discrepancies using the coefficients and methods described by Junchang and Masek^49^. The received NDVI images were composited into annual mosaics by taking the maximum value of each pixel. Based on the annual mosaics, we calculated the NDVI index for the studied plots in each year as an average pixel value within a plot (see Table S1 for value ranges observed). Such an approach is reasonable because of the relatively small size of breeding habitat patches (study plots) and the relatively high breeding pair density, which causes birds to not use a space limited to their territory but exploit the whole habitat patch (L. Gustafsson, personal observation). Thus, we get a measure of environmental variation among study plots across the studied years. However, due to the lack of available satellite images or accurate data quality, some information on NDVI is missing for the considered years and plots. The data query, processing, and calculations were performed using the cloud-based geospatial platform Google Earth Engine^50^ and the *raster* package in the R environment, version 4.0.3^51,52^.

### Data selection

The dataset used in the study was first filtered to include only records of females. This is because data on males is less representative as females are caught easier (mostly in the nest during incubation) than males, thereby females’ life histories are more complete. More importantly, due to the occurrence of extra-pair paternity and polygyny in the studied population of the collared flycatcher, the data on age-dependent reproductive performance is less accurate for males compared to females. Moreover, males contribute poorly to such reproductive traits as laying date or clutch size. For this reason, the analyses were limited to females only. Next, we excluded from the dataset all the females who were involved in any experimental manipulations. In fact, since the early 1980s, a number of experiments have been performed on the Gotland population of collared flycatchers, mainly to examine the costs of an increased reproductive effort, the effects of parental investment in relation to differential rearing conditions, or the trade-offs between reproduction and immune function. A large part of those studies were primarily based on brood size manipulation, which can obviously modify reproductive performance not only in the year of a given experiment but also in subsequent years as a potential carry-over effect. Moreover, since our study is focused on the effects of environmental variation (NDVI) on age-specific patterns in reproductive performance, any additional environmental variation associated with brood size manipulation would disturb a reliable assessment of effects linked to NDVI. However, the mentioned experiments may affect not only focal females but also the next generation of females (i.e., daughters). Thus, to avoid the potential confounding effects of such experiments, we decided to eliminate the records of both such females and their daughters from our analyses (in total, about 66% of all females were rejected). Afterwards, we selected females that were ringed as nestlings (i.e., in the year of hatching) or as first-year breeders (i.e., yearlings) to get a dataset with females of an exact known age. Based on this, we determined the observed age at first reproduction (AFR) and the age at last reproduction (ALR) at which individuals appear or disappear in the studied population. To get a reliable determination of ALR, we removed females with gaps in their individual histories resulting from not detecting them in a given year during breeding season (4.3% of all females with an exact known age). This allowed us to avoid an unreliable determination of ALR. In the case of AFR, we removed 34 females that started to breed in the population at the age of four or later (1% of all females with an exact known age), which minimised a chance to operate on data confounded by overlooking potential episodes of previous breeding by such breeders. Due to small sample sizes, some age classes were pooled into one mutual class. Thus, the females involved in our dataset were classified into five groups according to ALR (at one, two, three, four, and equal or higher than five years of age). The selected data also contained information on laying date, clutch size, the number of produced offspring, and the number of recruits. Ultimately, the data used in our analyses included 2843 laying dates of 2058 females, 2822 clutch sizes of 2044 females, and 2422 broods with a determined number of fledglings and recruits of 1802 females. Sample sizes differed due to missing data on reproductive traits and NDVI measurements.

### Statistical analysis

To examine the effects of female age and environmental conditions (NDVI) on reproductive performance, we used a frequentist framework. We constructed linear mixed models (LMMs) with a Gaussian error distribution and an identity-link function to analyse laying date, clutch size, and offspring production (the number of fledglings) per female per year. In turn, a generalised linear mixed model (GLMM) with a Poisson error distribution and a log-link function was fitted to analyse the number of recruits per female per year. Further, GLMM with a binomial error variance and a logit-link function was fitted to analyse the probability of brood-level recruitment (i.e., whether any offspring from a brood was or was not recruited). In all models, we included age as both a linear and a quadratic term to test for non-linear variation in reproductive traits with age and a linear term of NDVI as a measure of environmental conditions (habitat quality). Additionally, as we were primarily interested in studying the within-female effect of age on reproductive traits, we introduced both AFR and ALR to our models. Such an approach allows for the control of the selective appearance and disappearance of individuals in a population and therefore provides a good solution to distinguish within- and between-individual effects^53,54^. To control for breeding date in clutch size analysis, maternal investment in clutch size in fledging number analysis, and the number of fledged offspring in recruitment analyses, we used residuals estimated from previously performed models instead of the original variables (i.e., residuals from the laying date model, clutch size model, and fledgling number model, respectively). Using residuals instead of the original variables ensures that the variation in female age and NDVI explained by a dependent variable does not overlap with the variation in female age and NDVI in subsequent models. All of the explanatory variables were z-standardised, with mean = 0 and unit = 1 SD, to receive a meaningful value for the intercept and to obtain coefficient estimates comparable among the predictor variables. To control for the non-independence of data and between-subject variation in the examined traits, female identity, plot identity, and year of breeding attempt were introduced as random factors in all the models. Since our focus was to test the interaction between female age (a linear and quadratic term) and environmental conditions (NDVI), we expanded our models to include interaction terms. Thus, besides main effects, our full (initial) model contained two-way interactions: linear age × NDVI and quadratic age × NDVI. The full model has the following form (Equitation 1):

$$\begin{aligned} Reproductive\,trait_{ijk} & \sim \mu + \alpha_{1} Age_{ijk} + \alpha_{2} Age^{2}_{ijk} + \alpha_{3} AFR_{i} + \alpha_{4} ALR_{i} + \alpha_{5} NDVI_{jk} \\ & \quad + \left( {\alpha_{6} Residual_{ijk} } \right) + \alpha_{7} Age_{ijk} NDVI_{jk} + \alpha_{8} Age^{2}_{ijk} NDVI_{jk} \\ & \quad + ind_{i} + plot_{j} + year_{k} + \varepsilon_{ijk} \\ \end{aligned}$$

where a reproductive trait of female *i* for study plot *j* and in year *k* is explained by age (*Age*_*ijk*_) and age-squared (*Age*^2^_*ijk*_) of female *i* for study plot *j* and in year *k*, age at first (*AFR*_*i*_) and last reproduction (*ALR*_*i*_) of female *i*, a measure of environmental conditions (habitat quality) (*NDVI*_*jk*_) for study plot *j* and in year *k*, residual from a previous model (not applicable for the laying date model) of female *i* for study plot* j* and in year *k*, and the interactive terms. To reduce full models and evaluate whether and which interactions contribute significantly to explaining the variation in reproductive performance, we used a backward selection procedure based on the sequential removal of the least significant terms from a model. Thus, we removed non-significant interactive terms if *P* > 0.10, simultaneously controlling for the fit of models based on the Akaike Information Criterion (AIC). The main effects and random factors always remained in the fitted models; only the interactions were subject to a selection procedure. For all reproductive traits, we present the results of analyses based on the final (reduced, if applicable) model.

We performed all our analyses in the R environment, version 4.0.3^52^. LMMs and GLMMs were fitted using the *lme4* package in R^55^. The statistical significance of fixed effects in LMMs and GLMMs was tested based on t- and z-statistics, respectively. We used the *insight* package in R^56^ to estimate variance for all model terms, i.e., fixed factors (pooled), each random factor, and the residual component. LMMs analysing laying date, clutch size, and offspring production were visually checked to ensure that the assumptions of normality and homoscedasticity of residuals were met. Moreover, GLMMs were checked for overdispersion of residuals and zero-inflation using the *DHARMa* package in R^57^. We did not find our data to be zero-inflated or overdispersed.

### Supplementary Information

Supplementary Information.

## Data Availability

The dataset used and analysed during the current study is available from the corresponding author on reasonable request.
