# Supplementary material for: Age- and trait-dependent breeding responses to environmental variation in a short-lived songbird
Source: Sci Rep. 2023 Sep 11;13:14967. doi: 10.1038/s41598-023-42166-2 (PMC10495331; doi:10.1038/s41598-023-42166-2)
Supplement: Supplementary file 1 — Supplementary Information. [file 41598_2023_42166_MOESM1_ESM.pdf]

SUPPLEMENTARY MATERIAL

Age- and trait-dependent breeding responses to environmental variation in a short-lived songbird

Martyka R., Arct A., Kotowska D., Gustafsson L.

Tables

Table S1. Characteristics of 24 study plots from which data were used in the current research. Study plot identity, a period of time for which data were analysed, approximate area in ha, the number of nest boxes in years 2002–2014 (before and after this period, the number of nest boxes may be different in most plots), habitat type (dense deciduous forest [deciduous forest], deciduous forest with sparse hay meadows [forest-meadow], and pine-dominated forest with admixture of deciduous trees [mixed forest]), and NDVI calculated per study plot (minimal and maximal values in the period of time for which data were analysed) are presented.

Study plot	Period	Approximate area (ha)	Number of nest boxes	Habitat type	NDVI values (range)
AN	1986-2016	11	61	Deciduous forest	0.56-0.97
BJ	1986-2016	8	63	Deciduous forest/Forest-meadow	0.46-0.97
BO	2003-2016	7	18	Forest-meadow	0.69-0.93
BS	1986-2016	18	91	Deciduous forest/Forest-meadow	0.47-0.94
FAI	1986-2016	7	47	Forest-meadow	0.58-0.95
FAII	1986-2016	6	18	Forest-meadow	0.65-0.96
FKI	1986-2016	17	126	Deciduous forest	0.57-0.96
FKII	2012-2016	1	10	Deciduous forest	0.76-0.92
FKIII	2012-2016	2	13	Deciduous forest	0.83-0.94
FOI	1986-2016	12	113	Deciduous forest	0.56-0.93
FOII	1986-2016	5	30	Deciduous forest	0.49-0.95
FP	1986-2016	11	159	Deciduous forest/Forest-meadow	0.49-0.97
GR	1986-2016	10	76	Deciduous forest	0.55-0.95
KT	2011-2016	4	20	Forest-meadow	0.68-0.95
OJ	1986-2016	24	179	Deciduous forest/Forest-meadow	0.51-0.96
OL	2011-2016	5	29	Deciduous forest	0.68-0.95
RM	2011-2016	6	36	Deciduous forest/Forest-meadow	0.86-0.94
RN	2011-2016	11	60	Deciduous forest/Forest-meadow	0.66-0.96
RO	1986-2016	11	77	Deciduous forest/Forest-meadow	0.62-0.95
RUE	1986-2016	12	51	Deciduous forest	0.57-0.94
RUWI	1986-2016	8	33	Deciduous forest/Forest-meadow	0.56-0.94
RUWII	1986-2016	15	45	Deciduous forest/Forest-meadow	0.55-0.95
TUI	1986-2016	22	86	Mixed forest	0.50-0.89
TUII	1986-2016	20	88	Mixed forest	0.53-0.91

Figures

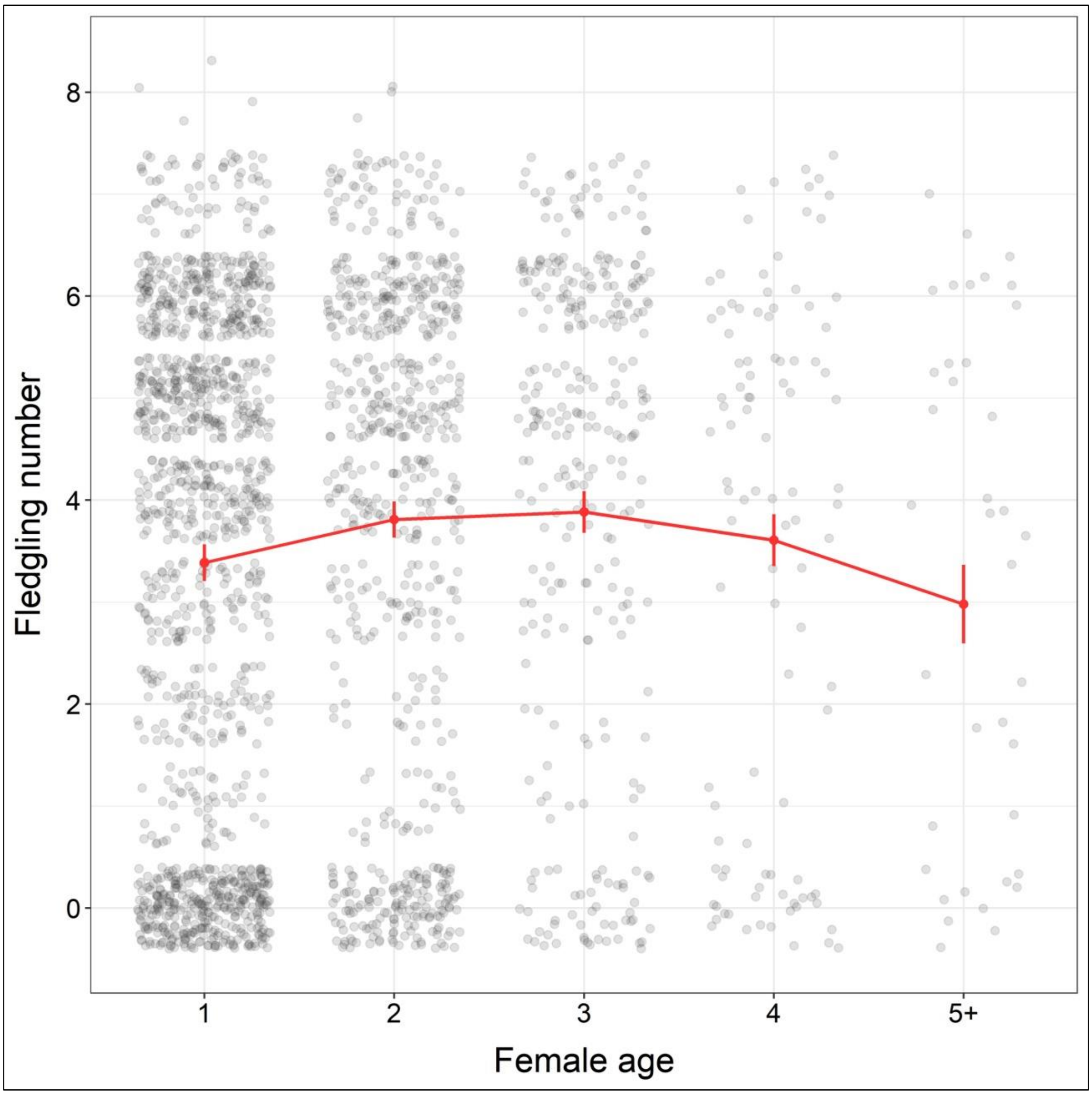

Figure S1. The quadratic effect of female age in relation to the number of fledglings. Predicted means with SEs (red) obtained from the final model and raw data points (grey circles) are presented.

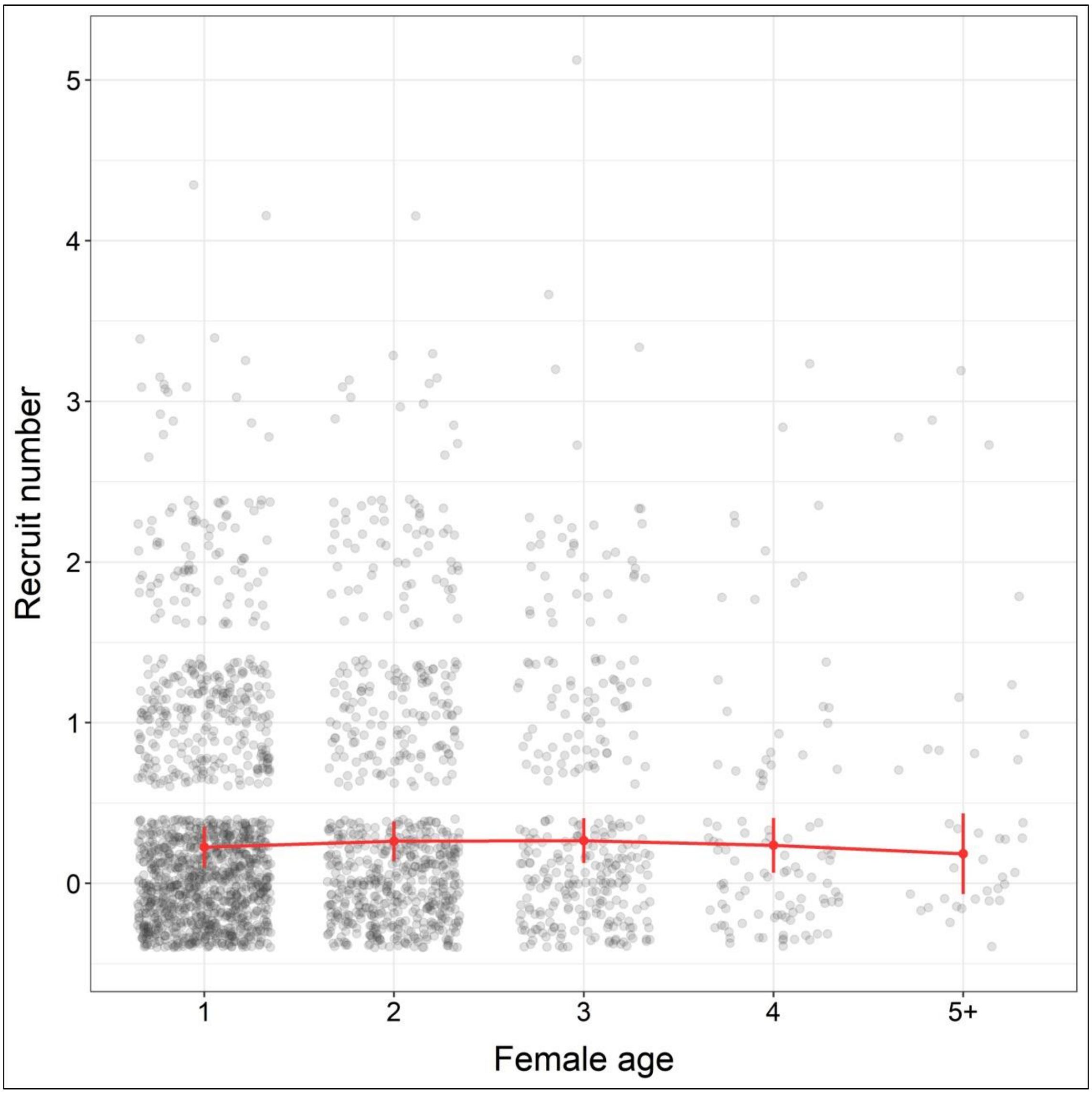

Figure S2. The quadratic effect of female age in relation to the number of recruits. Predicted means with SEs (red) obtained from the final model and raw data points (grey circles) are presented.
